# Folate and Colorectal Cancer in Rodents: A Model of DNA Repair Deficiency

**DOI:** 10.1155/2012/105949

**Published:** 2012-10-09

**Authors:** Rita Rosati, Hongzhi Ma, Diane C. Cabelof

**Affiliations:** Department of Nutrition and Food Science, Wayne State University, 410 W. Warren, 2018 Science Hall, Detroit, MI 48202, USA

## Abstract

Fortification of grains has resulted in a positive public health outcome vis-a-vis reduced incidence of neural tube defects. Whether folate has a correspondingly beneficial effect on other disease outcomes is less clear. A role for dietary folate in the prevention of colorectal cancer has been established through epidemiological data. Experimental data aiming to further elucidate this relationship has been somewhat equivocal. Studies report that folate depletion increases DNA damage, mutagenesis, and chromosomal instability, all suggesting inhibited DNA repair. While these data connecting folate depletion and inhibition of DNA repair are convincing, we also present data demonstrating that genetic inhibition of DNA repair is protective in the development of preneoplastic colon lesions, both when folate is depleted and when it is not. The purpose of this paper is to (1) give an overview of the data demonstrating a DNA repair defect in response to folate depletion, and (2) critically compare and contrast the experimental designs utilized in folate/colorectal cancer research and the corresponding impact on tissue folate status and critical colorectal cancer endpoints. Our analysis suggests that there is still an important need for a comprehensive evaluation of the impact of differential dietary prescriptions on blood and tissue folate status.

## 1. Introduction

Folate deficiency has been linked to a variety of pathologic conditions and cancers. Perhaps most notably, folate is required during pregnancy for normal development of the neural tube closure. Once the connection between reduced dietary folate consumption and neural tube defects (NTDs) was well established, the FDA mandated fortification of grain-based foods with folic acid. This mandate resulted in a >25% decrease in incidence of NTDs in the United States [1]. This fortification resulted in a slight bump in average serum folate levels in the United States from approximately 12 ng/mL to approximately 19 ng/mL [2]. Normal range for serum folate concentration in humans is 2.7–17 ng/mL [3]. Thus, folate fortification has resulted in a positive public health outcome for its intended population, women of childbearing age, through moderate increases in serum folate levels and significant reduction in NTD incidence. However, folate is also strongly connected through epidemiological data to an increased risk to develop colorectal cancer. Unlike prevention of NTDs which targets young, healthy populations, colorectal cancer is primarily a disease of aging. Concern for whether folate fortification may be detrimental in this population group is born from rodent studies demonstrating a potentially negative effect of folate supplementation on disease pathology. The purpose of this paper is to evaluate this concern with a particular focus on the impact of folate depletion and supplementation in rodent models. We have focused on data evaluating colorectal cancer phenotypes.

Folate is ingested from food, primarily from fruits and vegetables in the form of polyglutamated folate, and from folate supplements (primarily folic acid), and is ultimately metabolised into a variety of oxidized and reduced forms with varying levels of methylation, thoroughly reviewed elsewhere [4]. The different folate forms are essential for purine synthesis, methionine remethylation (and therefore S-adenosyl methionine metabolism), and thymidylate synthesis, all of which can play important roles in genomic stability. The work from Fenech's lab has been instrumental in establishing that chromosomal instability arises when folate is depleted, primarily in the form of micronuclei [5, 6]. Recently, Crasta et al. have demonstrated that micronuclei can induce further genomic instability through errors in chromosome segregation and genomic integration, as well as through chromothripsis, a process linked to carcinogenesis through massive chromosomal breakage and rearrangement [7]. These data provide a potentially direct mechanism for the carcinogenicity of folate depletion. 

Micronuclei originate from acentric chromosomes, chromatid fragments, or whole chromosomes that fail to attach properly to the mitotic spindle during anaphase and therefore do not segregate properly during cytokinesis [5]. Experimental data demonstrates that folate depletion causes micronuclei formation [8–10], and genetic data likewise establishes a role for folate metabolism in micronuclei formation. SNPs in the reduced folate carrier (RFC) gene (G80A), the methionine reductase (MTR) gene (A2756G), and the MTHFR gene (C677T and A1298C) are associated with the formation of micronuclei [11–13]. Interestingly, SNPs in several DNA base excision repair genes have also been associated with micronuclei formation: OGG1 (C1245G), TDG (G595A), and XRCC1 (C26304T, G26466A, and G28152A) [14]. We suggest that a reduced ability to fully repair uracil in DNA results in an accumulation of DNA damage that promotes strand breakage and micronuclei formation. 

## 2. Evidence That Folate Depletion Inhibits DNA Repair

Evidence collected from a variety of laboratories over the past decades has demonstrated an accumulation of DNA damage and/or mutations when folate is deficient. The mutagenic response to ENU (ethyl nitrosourea) is greater when folate is deficient [15]; EMS (ethyl methanesulfonate) and folate depletion induce a synergistic accumulation of DNA damage in Chinese hamster ovary (CHO) cells [16], and increased damage in response to MMS (methyl methane sulfonate) and hydrogen peroxide is seen in human colon epithelial cells when folate is depleted [17]; folate depletion makes human lymphocytes more sensitive to hydrogen peroxide [18], and more oxidative damage accumulates in response to amyloid *β*-peptide in neuronal cells depleted of folate [19]. These examples of accumulating damage point to an inability to repair the types of DNA damage repaired by the DNA base excision repair (BER) pathway. We directly tested the BER capacity of tissues exposed to oxidative DNA damage and found that folate depletion prevented induction of the BER pathway [20]. Further, we have shown that DNA strand breaks that arise in response to folate depletion accumulate to a larger degree when the BER pathway is genetically altered to have 50% reduction in capacity [21], demonstrating a direct role for the BER pathway in the DNA damage phenotypes of folate depletion.

**Figure 1:**
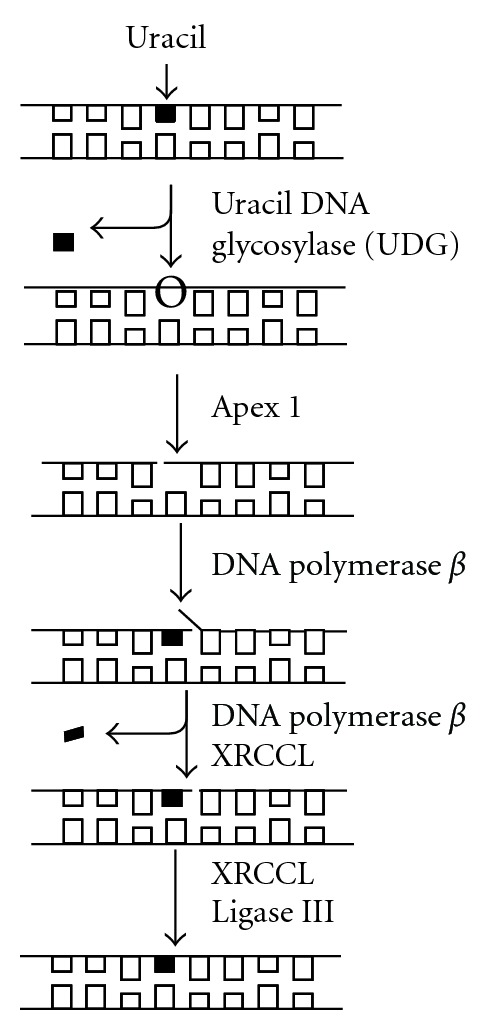
Biochemistry of base excision repair in uracil removal. Uracil removal is carried out as depicted, with initiation of removal by a uracil-excising DNA glycosylase (UDG depicted). All the uracil-excising glycosylases are monofunctional and leave behind an abasic lesion with an intact DNA backbone. An endonuclease (Apex 1) incises the DNA backbone 5′ to the abasic lesion, generating a 3′hydroxyl group and a 5′deoxyribose flap. A DNA polymerase (DNA polymerase *β*) inserts the correct nucleotide, then, in conjunction with a scaffolding protein (XRCCI), excises the deoxyribose flap. This step represents the rate-determining step in uracil-initiated base excision repair. Ligation of the scission in the phosphodiester backbone (ligase III and Xrcc1) completes repair and restores intact DNA structure. The single-strand break induced by Apex1 persists until ligation is complete and presents a potentially cytotoxic lesion if left incompletely repaired.

Folate deficiency has been shown to result in an accumulation of uracil in DNA, a BER substrate, likely through altered thymidylate synthesis and a resulting dUMP/TMP imbalance. Uracil is uniquely removed from DNA by the BER pathway in a DNA-polymerase-*β*-(*β*-pol-) dependent fashion. In response to uracil accumulation, BER is initiated by a uracil DNA glycosylase (predominantly Udg). The processing of uracil induces transient DNA strand breaks that are ultimately resolved as repair is completed, as described in Figure 1. As such, if folate deficiency inhibits the BER pathway specifically as we have shown, we should then expect the folate deficient phenotype to mimic that of BER deficiency. In addition to accumulating DNA single-strand breaks, mutation frequencies, and DNA damage sensitivity described above, these folate-specific phenotypes also include chromosome breakage, micronucleus formation, defects in chromosomal condensation, and expression of chromosomal fragile sites ([22–26]. Many of these same phenotypes are induced by deficient BER such that the phenotypes of folate depletion closely mimic the phenotypes of BER deficiency. In Table 1 we present the phenotypes expressed by BER mutants. These phenotypes include uracil accumulation; mutation induction, increased DNA base damage, increased levels of DNA single and double strand breaks, microsatellite instability, and increased levels of sister chromatid exchange (SCE) and chromosomal aberrations (referenced in Table 1 [20, 21, 27–62]). Our observation that folate depletion induced a phenotype very similar to that of BER depletion suggested to us that folate depletion might exert an inhibitory effect on activity of the BER pathway.

Accordingly, we have recently shown that the inhibitory effect of folate depletion on BER is achieved in part through inhibiting transactivation of the rate-limiting activity of BER, *β*-pol. Early work on the *β*-pol promoter clearly identified the CRE palindrome as being essential for ATF/CREB activation of the promoter [63]. We have identified a region within the *β*-pol CRE element that is blocked when folate is depleted, and prevents transactivation when folate is deficient [20]. Demonstrating direct inhibition of the BER response to DNA damage is important with respect to connecting the phenotypes of folate depletion and BER deficiency. We suggest that the BER inhibition of folate depletion is dependent on initiation of the BER response without completion of repair, resulting in a repair imbalance. This comes from reports that the clastogenic phenotypes of BER deficiency are wholly dependent on glycosylase-mediated initiation of BER [64]. 

### 2.1. Uracil as a Source of Imbalanced Base Excision Repair and Double-Strand Break Formation

Glycosylase-mediated induction of BER begins a series of enzymatic reactions that induces a break in the phosphodiester backbone; a break that persists until repair is completed (Figure 1). We have shown that folate depletion induces uracil DNA glycosylase (UDG) activity in liver without a corresponding induction of the rest of the pathway, generating an imbalance in BER and an accumulation of DNA single-strand breaks [21]. Others have likewise shown accumulation of single strand breaks and double strand breaks in response to folate depletion [65, 66]. Strand breaks set the stage for chromosomal instability including dicentric formation, anaphase bridges, and gross amplifications/deletions [67]. We propose that it is through these uracil-initiated strand breaks and chromosomal aberrations that folate promotes micronuclei formation, a likely carcinogenic precursor. Recently, MacFarlane et al. have demonstrated a key role for uracil misincorporation as a driving force in the colorectal carcinogenesis of folate depletion ([68]). Folate depletion and serine hydroxymethyltransferase heterozygosity (SHMT^+/−^) resulted in a twofold increase in number of colon tumors in the APCmin model of intestinal tumorigenesis. Moreover colonic DNA uracil content doubled when APC^min⁡/+^ SHMT^−/+^ mice were folate depleted, correlating to observed decreases in thymidylate synthesis protein abundance. SHMT uses serine as the one carbon donor to convert tetrahydrofolate (THF) to 5,10 methyleneTHF, the 1C donor for conversion of dUMP to TMP. These results directly connect the increased tumorigenesis observed in colons of APC^min⁡/+^ SHMT^−/+^ to changes in thymidylate synthesis and resultant uracil misincorporation into DNA.

## 3. Folate Depletion and Colorectal Carcinogenesis

Many epidemiological studies support the protective effect of folate in prevention of colorectal cancer. Most recently, a meta-analysis of 13 human studies shows a positive correlation between folate consumption and protection from colorectal cancer [69]. Accordingly, many rodent studies demonstrate that folate depletion increases tumorigenesis and/or the development of precursor lesions (aberrant crypt foci, ACF) in response to colon carcinogens. In the past decade, this protective effect of folate supplementation and detrimental effect of folate depletion have been called into question in response to several studies in which folate supplementation increased the numbers of tumors in tumor models of colorectal carcinogenesis, and correspondingly folate depletion reduced tumor development or aberrant crypt formation. These contradictory findings have been reviewed [70], and the short explanation is that folate supplementation is potentially detrimental during the promotion phase of carcinogenesis, either as a function of carcinogen exposure or genetic manipulation.

### 3.1. Analysis of Dietary Intervention Strategies and Impact of Folate Status and Intestinal Endpoints

Conclusions about the potential dangers of folate on colorectal cancer development may be based, in some instances, on unequal comparisons. A primary objective of this paper is to complete a careful analysis of dietary intervention studies to evaluate the importance that differences in model systems and/or dietary interventions may have on critical colorectal cancer endpoints. In Table 2, we have tabulated this information to allow facile comparison of dietary interventions across studies. We have included information on the following: animal model, diet source (when provided), length of dietary intervention, whether the study design included use of antibiotics to prevent microbial production of folates in the colon or use of wire-bottom caging to prevent coprophagy, and, the impact of the dietary intervention on folate levels (when provided). Included are studies that present data pertaining specifically to colorectal cancer endpoints, including mutation frequency, ACF, and intestinal tumors [71–81]. (These endpoints are presented in detail in Table 4). What we find is that the typical dietary intervention uses a dietary prescription of 2 mg/kg folate for the control group, with experimental groups of 0 mg/kg folate (deficient) and 8 mg/kg (supplemented). Some studies have used 20 mg/kg folate as a hypersupplemented group as well, which does not appear to confer additional advantage or disadvantage. Some studies have used 8 mg/kg folate as the control diet [73] and some have used 5 mg/kg folate as the control diet [82], which makes comparison across studies difficult. As standard chow diets provide on average 8–10 mg/kg folic acid, these study designs facilitate comparisons across studies in which undefined diets have been used. However, it does make interpretation of the data difficult within the context of folate dose on cancer risk. This is illustrated in the summary data we present (Table 3) on percent change in blood folate and colonic folate status in response to dietary intervention. Using 2 mg/kg folate as control, there is a 50–96% decrease in blood folate at 0 mg/kg folate (with the use of antibiotics or prevention of coprophagy), and a 58–140% increase at 8 mg/kg folate. This makes it difficult to interpret the role of folate depletion and/or supplementation when the comparison is between 8 mg/kg and 0 mg/kg folate. 

Duration of dietary intervention appears to affect the impact on folate status. Very few studies have measured colonic folate status in response to dietary depletion, which is the target tissue of interest for this paper. But in two papers in which this was determined there seems to be a significant impact of increasing the length of the study on colonic folate levels. After 8 weeks of feeding, rats exhibit a 35% decrease in colon folate levels [83], but after 20 weeks there is a 72% decline [75]. The data in mice seems odd, in that the colon folate levels are more depleted after 5 weeks of dietary intervention [68] than after 16 weeks [84], but these studies are confounded by alterations in choline and other B vitamins. Further, these mouse studies were carried out in the absence of methods for reducing assimilation of microbial-produced folates in the colon, and there is a possibility that with the extended intervention time (16 weeks) animals adapt to reduced dietary availability of nutrients through increased coprophagy.

There also seems to be a differential sensitivity to folate depletion between mice and rats. From the limited data available, mice appear to become severely depleted (>90% reduced blood folate) after 8 weeks of feeding [21], while rats are only about 50% depleted at 8 weeks [83], 1996, but >90% depleted by 11 weeks or more of dietary folate restriction [85]. This holds true for colonic folate status as well. However, whether this 3-week difference is meaningful is unclear. 

The information in Table 3 clearly demonstrates the importance of antibiotic and/or wire-bottom cages for inducing the most severe folate depletion. The average reduction in blood folate when antibiotics and/or wire-bottom cages are used is >80% (with the majority of studies showing >90%), while the absence of these factors induces a more modest 61% reduction. Further evidence for the importance of severe dietary restrictions to induce a meaningful decline in folate status is the finding that 32 weeks of feeding 0.5 mg/kg folate without antibiotics or wire-bottom cages resulted in no change in blood folate status [78]. It would be useful to have data on the impact of folate depletion on colonic folate status with and without contribution from microbially produced folates, but that information is not available without the confounding of choline deficiency [68] or riboflavin, B6, and B12 deficiencies [84]. A systematic evaluation of dietary factors on target tissue folate status would be informative.

### 3.2. Impact of Dietary Folate Restriction on Colorectal Cancer Endpoints

 These considerations aside, there is a definite impact of altering blood and tissue folate status on colorectal cancer endpoints. And these differences seem to be clearly dependent on the stage of cancer development. Table 4 outlines studies in which colorectal cancer endpoints have been analyzed in response to dietary folate manipulations. It is very clear that in rats exposed to DMH (dimethylhydrazine), ENU (ethyl nitroso-urea), or AOM (azoxymethane) that dietary folate depletion is detrimental if the diet is begun prior to carcinogen exposure. Rats exposed to DMH had a 70% increase in colon tumors [83] and a 66% increase in ACF [85] when folate was depleted in the diet. In response to AOM, the increase in aberrant crypt foci was more modest at 12% increase [86], likely a function of a less restrictive dietary intervention (no antibiotics or wire-bottom caging). Because colorectal cancer is believed to be initiated by mutations in key genes, we also analyzed a paper in which the mutagenic response to ENU was evaluated (not a colon carcinogen) and found that ENU increased mutant frequency 8-fold when folate was depleted [15]. However, a very different effect is seen when diet is begun after carcinogen exposure. With respect to ACF and tumor formation, when folate depletion began post-AOM exposure, a slight decrease in ACF and tumor diameter was seen in the folate depleted group (0 mg/kg foalte) and a 47% increase was seen in the folate supplemented group (8 mg/kg folate) [76], suggesting that presence of folate was permissive for ACF and tumor formation. With respect to colon adenocarcinomas, Le Leu et al. [73] found that the total number of adenocarcinomas was 3-fold higher in the 8 mg/kg folate group as compared to the 0 mg/kg group. Clearly in this example folate depletion was protective against AOM-induced colon adenocarcinomas and folate supplementation was detrimental, but it remains unclear how either of these groups would have compared to an adequate folate diet of 2 mg/kg folate.

 In mouse studies, data are confounded by genotype differences in models predisposed to develop gastrointestinal tumors, as well as other genetic manipulations devised to investigate the role(s) of certain pathways on colon tumorigenesis. In total we present 4 mouse studies showing a protective effect of folate on colon tumorigenesis, and 2 studies showing both detrimental and protective effects. Each study presents its own limitations preventing direct comparisons and solid conclusions. For example, in the APC^min⁡^ mouse, two studies have been completed that reach two different conclusions. In the MacFarlane et al. study [68], three *Shmt* genotypes were investigated, wildtype, heterozygous, and null mutants. The authors found an effect of diet on tumor number and size in the *Shmt* heterozygous mice only, showing a 2-fold increase in both variables when folate was deficient. However, in this study choline was also depleted, making it difficult to know the specific impact of folate on tumorigenesis. In contrast, Song et al. [77] found that at 3 months folate depletion was detrimental (more ACF and more ileal adenomas as compared to 2 mg/kg folate), but that by 6 months folate depletion was significantly protective against the development of ileal adenomas. Oddly, folate supplementation was also protective, with the 2 mg/kg folate group having the highest number of ileal adenomas. The protective effect of folate observed in the MacFarlane et al. was seen only in the *Shmt* heterozygous animals, which is to say that in the wildtype animals which provides the appropriate comparison to the Song et al. study, no effect of diet was seen on tumorigenesis. Of note, neither of these studies utilized either antibiotics or wire-bottom caging, such that the impact of the dietary intervention on tissue specific folate status was likely moderate. 

 In two studies using a different APC model, the APC1638N mouse, riboflavin, B6, and B12 deficiencies were investigated along with folate deficiency such that the conclusions are not specific to folate. Additionally, both these studies avoided use of antibiotics and wire-bottom caging, so the impact of dietary intervention on folate status was moderate (see Table 3). Interestingly, in the Liu et al. study, this moderate folate depletion resulted in an approximate 40% decline in colon folate status. The Ciappio study measured small intestinal folate levels and found no difference in response to dietary manipulation; findings that are somewhat counterintuitive. With respect to the critical endpoints, the Liu et al. study found an almost 2-fold increase in tumor incidence (50% compared to 91%) in response to folate (and other B vitamins) depletion. This corresponded to an increased number of tumors/animal and a slight increase in aberrant crypt numbers. Ciappio et al. investigated the impact of maternal diet on cancer in offspring. Mothers were depleted during pregnancy and weaning, then offspring were separated into vitamin B-sufficient-, deficient- and supplemented-groups. Notably, the folate level in the vitamin-B-deficient group was 0.5 mg/kg folate, which effectively resulted in no changes in serum or small intestinal folate. This suggests that changes observed in critical endpoints may not be due to folate status but rather to other B-vitamins. Nonetheless, they observe a strongly protective effect of B-vitamin supplementation on both tumor incidence and multiplicity (~60% reduction). Oddly, B-vitamin supplementation resulted in the lowest target tissue (small intestine) and blood folate levels making it difficult to interpret the data with respect to tissue folate status. However, it is noteworthy that the deficient group exhibited the worst tumor invasiveness of all groups, clearly an important endpoint. 

 In the only study to investigate tumorigenesis in response to folate depletion in a mouse strain other than C57bl/6, Knock et al. have shown that folate depletion increased the number of duodenal tumors in the BALB/c strain [81]. Unfortunately folate status was not determined, but the use of antibiotics suggests that these animals would have been significantly depleted. This was a relatively small number of animals (2/16 developed the duodenal tumors), but none of the mice in the control group developed tumors, so there is a definite effect in these animals as compared to the C57bl/6. The effect of strain differences in susceptibility to dietary intervention is potentially quite interesting. Studies have demonstrated significant copy number changes between strains, and even between substrains [87, 88], and these changes could potentially account for the observed differences. Along these lines, it's also important to consider the impact that multiple crossings into different strains (129 for making transgenics, e.g.) may have. Even with what is presumed to be adequate backcrossing, it's inevitable that some DNA sequence does not get fully backcrossed out. In this reviewer's opinion, this consideration has broad implications, but implications that also present opportunities for exploitation of copy number change-induced phenotype effects. To my knowledge, the only other study that has shown increased incidence of cancer in response to dietary folate manipulation was Pogribny and James [79], in which methyl donor deficiency resulted in liver tumors. To date, there are no studies showing that dietary folate (or methyl donor, or B-vitamin) deficiency induces colon tumors in rodents in the absence of carcinogen exposure. Even the genetic manipulations do not induce colon tumors, so data for answering this question is lacking.

 Two studies present data demonstrating both protective and detrimental effects of folate on critical endpoints. Song et al. [77] described above, found that at 3 months of age in the APC^min⁡^ mouse folate was protective, while at 6 months of age provision of folate was detrimental. These findings suggest that folate may help prevent initiation (3 months) while fueling tumor growth during promotion (6 months), consistent with the rat studies showing that folate drives tumorigenesis in the initiated colon. In the Ventrella-Lucente et al. paper [59], the impact of DNA base excision repair (BER) capacity on the tumorigenesis of folate depletion was investigated using a mouse model of BER deficiency [57]. This model develops lymphoma and adenocarcinoma in the absence of chemical exposure [58], suggesting that they would be more susceptible to the carcinogenic impact of folate deficiency. Using ACF as the critical endpoint, they demonstrated that folate depletion more than doubled the number of ACF in response to DMH exposure, consistent with the findings in rat models described above [83, 85]. However, in the BER mutant animals (DNA polymerase *β* heterozygotes), the reverse effect was seen on ACF formation. Here folate depletion significantly reduced the total number of ACF in response to DMH. DNA repair deficiencies are modifiers of penetrance [89], but typically in the opposite direction observed in the Ventrella-Lucente paper. However, another study looking at the APC^min⁡^ mouse likewise found that a DNA repair mutant (Ku70) ameliorated the impact of the APC mutation on tumorigenesis [90]. This study did not investigate a role for folate, but demonstrates the counterintuitive impact that loss of DNA repair capacity can have on tumorigenesis.

## 4. Conclusions

It becomes clear that while each study presents important information regarding the impact of folate on genomic stability in the colon, that there is some problem with a lack of consistency across study designs that prevent us from arriving at definitive conclusions. As the body of literature on folate continues to grow, these gaps in knowledge will be filled. We suggest that there is still an important need for a comprehensive study investigating the impact of differential folate prescriptions on blood and tissue folate status. We have shown here that the duration of feeding, dosage of folate, and use or avoidance of antibiotics and/or wire-bottom caging all impact the severity of folate depletion. Another point to consider is the difference in total blood folate levels between rodents and humans. The normal range for serum folate in humans is 2.7–17 ng/mL, manyfold lower than the average values observed in mice and rats. The range for mouse values reported in Table 2 is 39–170 ng/mL, for an average of 82.5 ng/mL, or about 8-fold higher than human values. The absolute values for rats reported in Table 2 range from 34 to 684 ng/mL. Excluding the two very high values (657 and 684 ng/mL), the average rat blood folate level is 60 ng/mL—6-fold greater than human. In essence, the dietary regimens that we consider to be folate depleted in rodent studies seem only to bring rodent levels to within normal limits for human values, and then only when the most severe diets are utilized. This is an observation that should be duly considered when theoretically extrapolating mouse data to human diseases. However, with respect to expressed phenotypes within one model system we can confidently state these to be a function of folate status, regardless of how these values compare to folate levels in independent model systems. One last point to be made is that fortification of grain products is not analogous to the folate supplementation regimes used in these animal studies. Fortification of grain products with folic acid is intended to prevent deficiency of folate, and this public health action has effectively increased average folate levels from 12 ng/mL to approximately 19 ng/mL [2]. This increase is still far less than the 60 ng/mL (rat) and 80 ng/mL (mouse) levels attained on the maintenance (2 mg/kg) diets, such that fears of detrimental effects of folate fortification are unwarranted. That is, folate fortification is not folate supplementation and concerns that fortification of grains with folic acid is dangerous are unsupported by data in the literature.

## Figures and Tables

**Table 1:** Genome instability phenotypes in base excision repair mutant models.

Gene	Genotype	Phenotype	Genome instability
UNG [27–29]	Ung^−/−^	Viable B-cell lymphoma Neuronal sensitivity to oxidative damage Neurodegeneration	Uracil accumulation in brain

SMUG [30, 31]	Smug^tg/+^ Smug^/siRNA^Ung^−/−^	Viable	C to T mutagenesis

OGG1 [32–35]	Ogg1^−/−^	Viable UVB-induced skin tumors	8-OHdG accumulation G to T mutagenesis Gamma radiation-induced DSB in OGG1 overexpressing cells

MYH [36, 37]	Myh^−/−^ Ogg1^−/−^Myh^−/−^	Viable Reduced survival Lung, ovarian, and lymphoid tumors	Spontaneous mutagenesis G to T mutagenesis

AAG [38–40]	Aag^−/−^	Viable Sensitivity to alkylation damage Retinal degeneration in +/−	Increased mutagenesis

NTH			
[41, 42] [35, 43]	Nth1^−/−^ Ogg1^−/−^Nth^−/−^ TK6 cell line	Viable	Increased thymine glycol in liver after X-ray irradiation
[44]	Ogg1^−/−^Nth^−/−^ mice	Viable	Gamma irradiation-induced DSB H_2_O_2_ resistant

TDG [45]	Tdg^−/−^	Embryonic lethal	Deficient repair of mtDNA

MBD4 [46, 47]	Mbd4^−/−^ Mbd4^−/−^Apc^min/+^	Viable Intestinal adenomas	Aberrant chromatin metabolism C to T mutagenesis

FEN [48, 49]	Fen1^−/−^ Fen^+/−^Apc^1638N^	Early embryonic lethal Intestinal adenocarcinoma Decreased survival	Microsatellite instability Extensive apoptosis

APE [50, 51] [20]	Ape^−/−^ Ape^+/−^	Embryonic lethal Sensitive to oxidative stress	
[52]	Apex1^+/−^XPC^−/−^	Increased UV-induced skin cancer	Increased mutagenicity Papillary adenocarcinoma and lymphoma

XRCC [53, 54]	Xrcc1^−/−^	Embryonic lethal	
[55, 56]	Xrcc1^+/−^	Increased AOM-induced ACF	SCE in embryo and cell lines

*β* -Pol [21, 64]	*β*-Pol^−/−^	Embryonic lethal	DSB accumulation Increased mutagenesis
[57–60]	*β*-Pol^+/−^	Viable Accelerated aging Lymphoma and adenocarcinomas	Increased SCE in MEFs SSB accumulation

LIGI [61]	Lig^−/−^	Embryonic lethal defective erythropoiesis	Oxidative stress sensitivity Increased mutagenesis Chromosomal aberrations

LIGII [62]	Lig^−/−^	Embryonic lethal	Elevated SCE

**Table 2:** Impact of experimental design on blood and tissue folate.

Animal model	Experimental diet	Abx	Wire cages	Length of feeding	In vivo folate levels	Citation
Rat studies quantifying impact of dietary intervention on blood and/or tissue folate status						

Sprague-Dawley rats	Amino acid defined (Dyets) *Control group*: 8 mg/kg folate *Experimental group*: 0 mg/kg folate	No	Yes	25 weeks	Folate levels (nmol/g) *Control group: * Liver 27.11 Kidney 11.69 Spleen 3.74 Brain 0.65 *Experimental group: * Liver 11.10 Kidney 4.79 Spleen 1.29 Brain 0.60	[71]

Sprague-Dawley rats	Amino acid defined (Dyets) *Control group*: 2 mg/kg folate *Experimental groups*: 0 mg/kg folate 8 mg/kg folate 20 mg/kg folate	No	Yes	8 weeks	*Control group*: Plasma folate (ng/mL)~50 Colonic folate (ng/mL)~650 *Experimental groups*: Deficient Plasma folate (ng/mL)~25 Colonic folate (ng/mL) ~480 Supplemented (8 mg/kg) Plasma folate (ng/mL) ~80 Colonic folate (ng/mL) ~975 Supplemented (20 mg/kg) Plasma folate (ng/mL) ~140 Colonic folate (ng/mL) ~975	[83]

Sprague-Dawley rats	AIN-76 semipurified diet *Control group: * 8 mg/kg folate, no abx *Experimental groups: * 0 mg/kg folate, no abx 0 mg/kg folate, with abx first 4 weeks of feeding 0 mg/kg folate, with abx last 4 weeks of feeding	Yes and no	Yes	12 weeks	Whole blood folate *Control group: * 657 ng/mL *Experimental group: * 125 ng/mL (no abx) 114 ng/mL (abx first 4 weeks) 61 ng/mL (abx last 4 weeks)	[72]

Sprague-Dawley rats	AIN-76 semipurified diet *Control group: * 8 mg/kg folate, no abx *Experimental groups: * 0 mg/kg folate, no abx 0 mg/kg folate, with abx first 4 weeks of feeding 0 mg/kg folate, with abx 4 weeks after AOM	Yes and no	Yes	26 weeks	Whole blood folate *Control group: * 684 ng/mL *Experimental groups: * 694 ng/mL (control + abx) 99 ng/mL (0 mg/kg, no abx) 100 ng/mL (0 mg/kg, abx first 4 weeks) 96 ng/mL (0 mg/kg, abx 4 weeks after AOM) Colon folate *Control group: * 9.6 ug/mg *Experimental groups: * 8.0 ug/mg (control + abx) 2.7 ug/mg (0 mg/kg, no abx) 3.1 ug/mg (0 mg/kg, abx first 4 weeks) 4.3 ug/mg (0 mg/kg, abx 4 weeks after AOM)	[73]

Sprague-Dawley rats	AIN93 (G or M not Specified) *Control group: * 2 mg/kg folate *Experimental group: * 0 mg/kg folate	No	No	20 weeks	Hepatic folate (nmol/g) *Control group: * 17 nmol/g (weanling) 17 nmol/g (12 month old) *Experimental group: * 2.7 nmol/g (weanling) 1.8 nmol/g (12 month old)	[74]

Sprague Dawley rats	AIN93 purified diet (G or M not specified) *Control group * 2 mg/kg (folate-replete) *Experimental groups: * 0 mg/kg folate 8 mg/kg	No	Yes	20 weeks	Plasma folate Young Old *Control group: * (umol/L) 34.1 30.6 *[(ng/mL) 15.1 13.5] * *Experimental group: * Deficient (umol/L) 1.5, 0.9 *[(ng/mL) 0.7 0.04] * *Supplemented: * (umol/L) 53.9 55.5 *[(ng/mL 23.8 24.6] * Colon (nmol/g) Young Old Control 4.7 3.0 Deficient 1.3 0.7 Supplemented 5.6 4.4	[75]

Fischer-344 rats	AIN-93 diet *Control group: * 2 mg/kg folate *Experimental group: * 0 mg/kg folate Note: selenium assessed in this paper, not evaluated here.	No	Yes	11 weeks	Plasma folate *Control group: * 277.4 nmol/L *[122.44 ng/mL] * *Experimental group: * 12.0 nmol/L *[5.30 ng/mL] *	[85]

Sprague-Dawley rats	Amino acid defined (Dyets) All animals fed same diet (2 mg/kg folate) for first 10 weeks; 5 weeks following completion of carcinogen exposure, experimental diets began. *Control group: * 2 mg/kg folate *Experimental groups: * 0 mg/kg folate 5 mg/kg folate 8 mg/kg folate	Yes	No	24 weeks	Plasma folate *Control group: * 32.2 ng/mL *Experimental groups: * Deficient 6.0 ng/mL Supplemented (5 mg/kg) 72.8 ng/mL Supplemented (8 mg/kg) 78.2 ng/mL Hepatic folate *Control group: * 7.7 ug/g *Experimental groups: * Deficient 5.0 ug/g Supplemented (5 mg/kg) 8.5 ug/g Supplemented (8 mg/kg) 9.9 ug/g	[76]

Male Hooded-Lister rats	AIN-93G purified diet with vitamin-free casein *Control group: * 5 mg/kg folate *Experimental group: * 0 mg/kg folate	No	Yes	6 weeks	Folate value, ng/mg protein Lymphocytes Control: 0.45 Experimental: 0.27 Liver Control: 136.2 Experimental: 93.9 Colon (descending) Control: 24.6 Experimental: 9.9 Spleen Control: 16.5 Experimental: 7.9 Kidney Control: 58.6 Experimental: 26.6 Brain Control: 20.4 Experimental: 15.3 Heart Control: 9.2 Experimental: 3.4	[82]

Mouse studies quantifying impact of dietary intervention on blood and/or tissue folate status						

C57bl/6J mice, APC^Min^	Amino acid defined (Dyets) *Control group: * 2 mg/kg folate *Experimental group: * 0 mg/kg folate 8 mg/kg folate* 20 mg/kg folate	No	No	3 months; 6 months	Serum folate (ng/mL): *Control group: * 39.0 (3 months) 35.4 (6 months) *Experimental group: * Depleted 12.1 (3 months) 10.8 (6 months) Supplemented 56.0 (3 months) 46.0 (6 months) Hypersupplemented 49.9 (3 months) 43.3 (6 months)	[77]

C57bl/6 mice, *β*-pol^+/−^	AIN-93G purified diet with vitamin-free casein *Control group: * 2 mg/kg folate *Experimental group: * 0 mg/kg folate	Yes	No	8 weeks	Serum folate *Control group: * 60 ng/mL *Experimental group: * <5 ng/mL	[21]

C57bl/6 mice, Aag^−/−^	AIN-93G purified diet with vitamin-free casein *Control group: * 2 mg/kg folate *Experimental group: * 0 mg/kg folate	Yes	Yes	4 weeks	Liver folate *Control group * 27.0 *μ*g/g *Experimental group * 1.2 *μ*g/g	[39]

					*Apc * ^ +/+^ * crossed to SHMT genotype as indicated * ^(+/+, +/−, −/−)^ *Control group: (5 weeks) * Plasma (ng/mL) 58.56 (+/+) 58.34 (+/−) 40.82 (−/−) Liver (fmol/ug pro) 51.80 (+/+) 56.65 (+/−) 50.77 (−/−)	
C57bl/6 mice, *Apc * ^min/+^ Shmt1 (^+/−^ and ^−/−^)	AIN-93G purified diet *Control group: * 2 mg/kg folate 2.5 g/kg choline *Experimental group: * 0 mg/kg folate 0 g/kg choline	No	Yes	5 weeks (Apc^+/+^); 11 weeks (Apc^min/+^)	Colon (fmol/ugpro) 35.14 (+/+) 21.46 (+/−) 17.09 (−/−) *Experimental group: (5 weeks) * Plasma (ng/mL) 20.60 (+/+) 38.95 (+/−) 8.52 (−/−) Liver (fmol/ug pro) 47.26 (+/+) 44.30 (+/−) 48.88 (−/−) Colon (fmol/ugpro) 9.15 (+/+) 18.04 (+/−) 14.89 (−/−) *Apc * ^min/+^ * crossed to Shmt genotype indicated * ^(+/+, +/−, −/−)^ *Control group: (11 weeks) * Plasma (ng/mL) 24.68 (+/+) 20.91 (+/−) 26.44 (−/−) Liver (fmol/ug pro) 45.72 (+/+) 40.00 (+/−) 41.50 (−/−) *Experimental group: (11 weeks) * Plasma (ng/mL) 11.79 (+/+) 8.37 (+/−) 9.97 (−/−) Liver (fmol/ug pro) 28.49 (+/+) 23.25 (+/−) 29.44 (−/−)	[68]

C57bl/6J mice, Shmt (^+/−^ and ^−/−^)	AIN-93G purified diet *Control group: * 2 mg/kg folate 2.5 g/kg choline *Experimental group: * 0 mg/kg folate 0 g/kg choline	No	No	32 weeks	*Control group: * Plasma folate (ng/mL) (wt) 36.3 (Tg) 46.8 Liver folate (fmol/ug pro) (wt) 43.1 (Tg) 51.3 *Experimental group: * Plasma folate (ng/mL) (wt) 7.4 (Tg) 5.5 Liver folate (fmol/ug pro) (wt) 36.1 (Tg) 34.0	[68]

C57bl/6 mice, APC^1638N^	Amino acid defined (Dyets) *Control group: * 2 mg/kg folate B-vitamin adequate *Experimental group: * 0 mg/kg folate B12, B6, and riboflavin deficient	No	No	16 weeks	*Control group: * Plasma folate (ng/mL) ~170 Colon folate (ng/g) ~500 *Experimental group: * Plasma folate (ng/mL) ~110 Colon folate (ng/g) ~300	[84]

C57bl/6 mice, APC^1638N^	*Maternal diet: * AIN93M (Dyets) Control and experimental *Offspring diet: * AIN93G during first 16 weeks of life AIN93M during last 16 weeks of life (Dyets) Control and experimental *Control group: * 2 mg/kg folate 6 mg/kg riboflavin 7 mg/kg B6 50 ug/kg B12 *Experimental groups: * Deficient 0.5 mg/kg folate 2 mg/kg riboflavin 2 mg/kg B6 10 ug/kg B12 Supplemented 8 mg/kg folate 24 mg/kg riboflavin 28 mg/kg B6 200 ug/kg B12	No	No	*Maternal diet: * Fed 4 weeks preconception through weaning *Offspring diet: * 32 weeks	Maternal *Control group: * Plasma folate (ng/mL) 84.7 Hepatic folate (ug/g) 13.2 *Experimental group: * Deficient Plasma folate (ng/mL) 81.4 Hepatic folate (ug/g) 11.1 Supplemented Plasma folate (ng/mL) 104.4 Hepatic folate (ug/g) 13.3 Offspring *Control group: * Plasma folate (ng/mL) 52.5 Hepatic folate (ug/g) 14.1 Sm Int folate (ng/g) 1205.9 *Experimental group: * Deficient Plasma folate (ng/mL) 59.3 Hepatic folate (ug/g) 12.9 Sm Int folate (ng/g) 1264.7 Supplemented Plasma folate (ng/mL) 50.2 Hepatic folate (ug/g) 13.3 Sm Int folate (ng/g) 1172.4	[78]

Folate depletion studies presenting critical colorectal cancer endpoints, but without folate status information						

Fisher 344 rats	AIN93G *Control group: * 2 mg/kg folate, −abx *Experimental groups: * 0 mg/kg folate, −abx 0 mg/kg folate, +abx	Yes and no	No	5 weeks	ND	[15]

Fisher 344 rats	NIH-31 *Control group: * 0.4% methionine 0.3% choline 2 mg/kg folate *Experimental group: * Low methionine 0% choline 0 mg/kg folate	No	No	36 weeks 54 weeks	ND	[79]

C57bl/6J mice	Casein/soy based *Control group: * 2 mg/kg folate *Experimental group: * 0 mg/kg folate	No	No	10 weeks	ND	[80]

BALB/cAnNCrlBR mice	Amino acid defined (Harlan Teklad) *Control group: * 2 mg/kg folate *Experimental group: * 0.3 mg/kg folate	Yes	No	12 to 14 months	ND	[81]

C57bl/6 mice Bpol^+/−^	AIN93G (Dyets) *Control group: * 2 mg/kg folate *Experimental group: * 0 mg/kg folate	Yes	No	12 weeks total (6 pre-DMH; 6 post-DMH)	ND	[59]

Albino rats	AIN93M *Control group: * 2 mg/kg folate *Experimental groups: * 8 mg/kg folate 40 mg/kg folate	No	No	6 weeks total (4 weeks pre-AOM; 2 weeks post-AOM)	ND	[86]

Values in brackets [] have been calculated from published values for ease of comparison across studies; +/+, +/− and −/− refer to wildtype, heterozygous and null genotypes; ND: not determined.

**Table 3:** Impact of dietary intervention on blood and colon folate status.

Percent change in blood folate status by dietary intervention			
2 mg/kg to 0 mg/kg	↓96%	Rat	20 wk
(with either abx or wire bottom cages)	↓96%	Rat	11 wk
	↓92%	Mouse	8 wk
[21, 75, 76, 83, 85]	↓50%	Rat	8 wk
	↓81%	Rat	24 wk

2 mg/kg to 0 mg/kg	↓69%	Mouse	12 wk
(without abx or wire bottom cages)	↓63%*	Mouse	5 wk
	↓78%*	Mouse	11 wk
[68, 77, 84]	↓35%**	Mouse	16 wk

2 mg/kg to 8 mg/kg	↑58%	Rat	20 wk
(with either abx or wire bottom cages)	↑62%	Rat	8 wk
[75, 76, 83]	↑140%	Rat	24 wk

2 mg/kg to 8 mg/kg			
(without abx or wire bottom cages)	↑44%	Mouse	12 wk
[77]			

Percent change in colon folate status by dietary intervention			

2 mg/kg to 0 mg/kg	↓72%	Rat	20 wk
(with either abx or wire bottom cages)	↓35%	Rat	8 wk
[75, 83]			

2 mg/kg to 0 mg/kg	↓74%*	Mouse	5 wk
(without abx or wire bottom cages)	↓40%**	Mouse	16 wk
[68, 84]			

2 mg/kg to 8 mg/kg	↑19%	Rat	20 wk
(with either abx or wire bottom cages)	↑66%	Rat	8 wk
[75, 83]			

2 mg/kg to 8 mg/kg			
(without abx or wire bottom cages)			

Abx: antibiotics; wk: week; *choline also depleted in this dietary intervention; **riboflavin, B6, and B12 also modified in this dietary intervention.

**Table 4:** Impact of experimental design on critical colorectal cancer endpoints.

Animal model	Carcinogen	CRC-specific endpoints measured
Studies demonstrating beneficial effects of folate on critical colorectal cancer endpoints		
Rat Sprague-Dawley Male [83]	DMH 44** **mg/kg body weight Weekly × 15 weeks	*Percent of rats with colonic tumors*: 70% (0 mg/kg folate) **40% (2 mg/kg folate)** 10% (8 mg/kg folate) 42% (40 mg/kg folate) (Similar results for # tumors/rat)

Rat Fisher 344 Male [15]	5-week diet prior to DMH ENU 100mg/kg	*Mutant frequency*: 8-fold increase (0 mg/kg folate, no antibiotics) 6-fold increase (0 mg/kg folate, with antibiotics) **5-fold increase (2 mg/kg folate)**

Rat Fisher 344 Male [85]	DMH 25** **mg/kg body weight 2 weekly injections	*Colonic aberrant crypts/aberrant crypt foci*: **~150 aberrant crypts, 50 foci (2 mg/kg folate)** ~250 aberrant crypts, 75 foci (0 mg/kg folate)

Rat Albino Male [86]	3-week diet prior to DMH 8-week diet after DMH AOM 30 mg/kg body weight	*Aberrant crypt foci*: **~65 aberrant crypts (2 mg/kg folate)** ~58 aberrant crypts (8 mg/kg folate) ~30 aberrant crypts (40 mg/kg folate) (note: no deficient group)

Mice Balb/cAnNCrlBR Wildtype (from 129) Backcrossed >10 generations into Balb/c Sex: not stated [81]	None, diet only	*Percent mice with duodenal tumors*: **0% (2 mg/gk folate)** 12.5% (0.3 mg/kg folate) (2/16 mice; adenoma versus adenocarcinoma not specified)

Mice C57bl/6 Apc1638N, BAT-LacZ (Wnt reporter mouse) No. of generations backcrossed N/A Sex: not stated [84]	None, diet and genotype only (Note: diet includes multiple B vitamin manipulations: Riboflavin, B6, B12, and folate)	*Gastrointestinal tumor incidence and multiplicity; aberrant crypt foci*: **50% incidence (2 mg/kg folate, adequate Bvitamins)** 91% incidence (0 mg/kg folate, B vitamin deficient) **~1.7 tumors/animal (2 mg/kg folate, adequate Bvitamins)** ~2.7 tumors/animal (0 mg/kg folate, B vitamin deficient) **~2 aberrant crypts (2 mg/kg folate, adequate Bvitamins)** ~2.5 aberrant crypts (0 mg/kg folate, B vitamin deficient)

Mice C57bl/6 Apc1638N No. of generations backcrossed N/A Sex: both [78]	None, diet and genotype only (Note: diet includes multiple B vitamin manipulations: Riboflavin, B6, B12, and folate; dietary intervention in dams and offspring)	*Gastrointestinal tumor incidence (percent) and multiplicity*: ~55% incidence (0.5 mg/kg folate, B vitamin deficient) **~58% incidence (2.0 mg/kg folate, Bvitamin adequate)** ~20% incidence (8.0 mg/kg folate, B vitamin supplemented) ~0.6 tumors/animal (0.5 mg/kg folate, B vitamin deficient) **~0.6 tumors/animal (2.0 mg/kg folate, Bvitamin adequate)** ~0.25 tumors/animal (8.0 mg/kg folate, B vitamin supplemented) (Note: tumor invasiveness significantly worse in deficient group compared to control group)

Mice C57bl/6 Apc^min^ >10 generations backcrossed Sex: not stated [68]	None, diet and genotype only Shmt heterozygous and null genotypes crossed onto Apc^min^ Note: choline altered as well as folate	*Gastrointestinal tumor number and tumor load (total tumor area/mouse)*: Impact of diet seen only in Shmt heterozygous model: **~32 small intestinal tumors (2 mg/kg folate, 2.5 g/kg choline)** ~60 small intestinal tumors (0 mg/kg folate, 0 g/kg choline) **~40 mm** ^2^ ** tumor load (2 mg/kg folate, 2.5 g/kg choline)** ~80** **mm^2^ tumor load (0 mg/kg folate, 0 g/kg choline)

Mice C57bl/6 DNA polymerase *β* ^+/−^ >10 generations Sex: Male [59]	DMH 30** **mg/kg body weight Weekly for 6 weeks Killed after 12 weeks	*Total aberrant crypt foci*: **~15 aberrant crypts (2 mg/kg folate, wildtype)** ~38 aberrant crypts (0** **mg/kg folate, wildtype) (Note: this work presents data both in support of protective and detrimental roles for folate; protective presented here, detrimental presented below)

Rat Sprague-Dawley Male [72]	AOM 15** **mg/kg Weekly for 3 weeks Killed after 8 weeks	*Total aberrant crypt foci*: ~300 aberrant crypts (8** **mg/kg folate) ~200 aberrant crypts (0** **mg/kg folate + abx post-AOM) (Note: no “standard” control group of 2** **mg/kg folate)

Rat Sprague-Dawley Male [73]	AOM 15 mg/kg/week Weekly for 3 weeks Killed after 22 weeks	*Number of colon adenocarcinomas*: 13 (8** **mg/kg folate, no abx) 12 (8** **mg/kg folate, with abx) 4 (0** **mg/kg folate, no abx) 3 (0** **mg/kg folate, abx before AOM) 4 (0** **mg/kg folate, abx after AOM) (Note: no “standard” control group of 2** **mg/kg folate)

Rat Sprague-Dawley Male [76]	AOM 2 weekly exposures (total dosing unclear) Diet begun 6 weeks post- AOM	*Aberrant crypt foci and tumor size (tumor diameter/tumor-bearing animal, cm) * 84.6 aberrant crypts (0** **mg/kg folate) **93.4 aberrant crypts (2 mg/kg folate)** 108.1 aberrant crypts (5** **mg/kg folate) 137.9 aberrant crypts (8** **mg/kg folate) 0.5 cm (0** **mg/kg folate) **1.2 cm (2 mg/kg folate)** 1.3 cm (5** **mg/kg folate) 1.6 cm (8** **mg/kg folate)

Mice C57bl/6 APC^min^ No. of generations backcrossed N/A Sex: not stated [77]	None, diet and genotype only Two timepoints: 3 and 6 months	*Aberrant crypt foci and ileal adenomas*: At 3 months: 1.3 aberrant crypts (0** **mg/kg folate) **0.27 aberrant crypts (2 mg/kg/folate)** 0.20 aberrant crypts (8** **mg/kg folate) 0.00 aberrant crypts (20** **mg/kg folate) 11.0 ileal adenomas (0** **mg/kg folate) **7.36 ileal adenomas (2 mg/kg folate)** 7.30 ileal adenomas (8** **mg/kg folate) 2.36 ileal adenomas (20** **mg/kg folate) At 6 months: 1.67 ileal adenomas (0 mg/kg folate) **7.09 ileal adenomas (2 mg/kg folate)** 5.33 ileal adenomas (8** **mg/kg folate) 4.38 ileal adenomas (20** **mg/kg folate)

Mice C57bl/6 DNA polymerase *β* ^+/−^ >10 generations Sex: male [59]	DMH 30** **mg/kg body weight Weekly for 6 weeks Killed after 12 weeks	*Total aberrant crypt foci: * **~33 aberrant crypts (2 mg/kg folate, heterozygote)** ~20 aberrant crypts (0** **mg/kg folate, heterozygote) (Note: this work presents data both in support of protective and detrimental roles for folate; detrimental presented here, protective presented above.)

~indicates values are approximated from graphical data; N/A: not available; Shmt: serine hydroxyl methyl transferase.
